# Advances in Nanomaterials and Colorimetric Detection of Arsenic in Water: Review and Future Perspectives

**DOI:** 10.3390/s24123889

**Published:** 2024-06-15

**Authors:** Abhijnan Bhat, Furong Tian, Baljit Singh

**Affiliations:** 1 School of Food Science & Environmental Health, Grangegorman, Technological University Dublin (TU Dublin), D07 ADY7 Dublin, Ireland; d21126441@mytudublin.ie (A.B.);; 2 Health, Engineering & Materials Science (HEMS) Research Hub, Technological University Dublin (TU Dublin), D24 FKT9 Dublin, Ireland; 3 Nanolab Research Centre, Physical to Life sciences Hub, Technological University Dublin (TU Dublin), D08 CKP1 Dublin, Ireland; 4 MiCRA Biodiagnostics Technology Gateway, Technological University Dublin (TU Dublin), D24 FKT9 Dublin, Ireland

**Keywords:** arsenic contamination, colorimetric sensing, nanomaterials, water

## Abstract

Arsenic, existing in various chemical forms such as arsenate (As(V)) and arsenite (As(III)), demands serious attention in water and environmental contexts due to its significant health risks. It is classified as “carcinogenic to humans” by the International Agency for Research on Cancer (IARC) and is listed by the World Health Organization (WHO) as one of the top 10 chemicals posing major public health concerns. This widespread contamination results in millions of people globally being exposed to dangerous levels of arsenic, making it a top priority for the WHO. Chronic arsenic toxicity, known as arsenicosis, presents with specific skin lesions like pigmentation and keratosis, along with systemic manifestations including chronic lung diseases, liver issues, vascular problems, hypertension, diabetes mellitus, and cancer, often leading to fatal outcomes. Therefore, it is crucial to explore novel, cost-effective, and reliable methods with rapid response and improved sensitivities (detection limits). Most of the traditional detection techniques often face limitations in terms of complexity, cost, and the need for sophisticated equipment requiring skilled analysts and procedures, which thereby impedes their practical use, particularly in resource-constrained settings. Colorimetric methods leverage colour changes which are observable and quantifiable using simple instrumentation or even visual inspection. This review explores the colorimetric techniques designed to detect arsenite and arsenate in water. It covers recent developments in colorimetric techniques, and advancements in the role of nanomaterials in colorimetric arsenic detection, followed by discussion on current challenges and future prospects. The review emphasizes efforts to improve sensitivity, selectivity, cost, and portability, as well as the role of advanced materials/nanomaterials to boost the performance of colorimetric assays/sensors towards combatting this pervasive global health concern.

## 1. Introduction

Arsenic contamination in water sources poses a significant threat to public health and the environment. It is a global concern, with millions of people exposed to toxic levels of arsenic through drinking water, primarily in regions where natural geological processes release this hazardous element into groundwater [1,2,3,4]. Arsenic occupies the top place on the 2022 priority list of the Agency for Toxic Substances and Disease Registry (ATSDR) [5]. Long-term exposure to arsenic can lead to severe health issues, including skin lesions, various cancers, and cardiovascular diseases. To tackle arsenic contamination and its toxicity, the WHO, US EPA, and EU have recommendations, laws, and directives that establish arsenic content limits in drinking, surface, and ground water. Furthermore, due to the harmful potential of inorganic As, the European Food Safety Authority (EFSA) recommends limiting its consumption, and the European Commission has established maximum amounts that can be same for consumption [6,7]. The WHO, EU, and US EPA maximum limit for arsenic in drinking water is 10 ppb [8,9,10]. Hence, there is an urgent need for reliable, cost-effective, and sensitive methods for arsenic detection in water.

While established spectroscopic techniques (Atomic Absorption Spectroscopy, Atomic Fluorescence Spectroscopy, Inductively Coupled Plasma Atomic Emission Spectrometry, and Inductively Coupled Plasma Mass Spectrometry) ensure accurate arsenic determination, several factors limit their practicality for widespread, real-time analysis. These techniques often necessitate laboratory-grade instrumentation, which can be both bulky and costly. Their operation and maintenance demand specialized knowledge and training, creating a barrier for routine deployment outside well-equipped facilities. Additionally, the sample preparation and analysis process for these methods can be time-consuming, further restricting their suitability for rapid, in-field assessments of arsenic contamination. These limitations underscore the demand for alternative solutions optimized for rapid, on-site analysis with minimal instrumentation, simpler operating procedures, and reduced costs [11,12,13,14,15,16,17]. In recent years, nanomaterials have emerged as promising candidates for the development of efficient and practical sensing platforms for the colorimetric detection of arsenic in water. Nanomaterials, characterized by their unique physical and chemical properties at the nanoscale, have garnered significant interest for their potential applications in sensor development [18]. These materials exhibit remarkable properties, including high surface area-to-volume ratio, tuneable optical properties, and enhanced reactivity, which can be harnessed to design sensitive and selective detection platforms. This review article explores the rapidly expanding field of nanomaterial-based colorimetric arsenic detection, summarizing the latest developments and highlighting the advantages and challenges associated with these novel approaches. The key motivation behind using nanomaterials for colorimetric arsenic detection is their ability to enhance the sensitivity and selectivity of traditional detection methods. Nanomaterials can be tailored to interact specifically with arsenic ions, resulting in a visible colour change that is easy to interpret, even in resource-limited settings. Furthermore, the use of nanomaterials offers the potential for on-site, real-time monitoring of arsenic contamination, which is essential for preventing long-term exposure and mitigating health risks [19,20,21].

**Figure 1:**
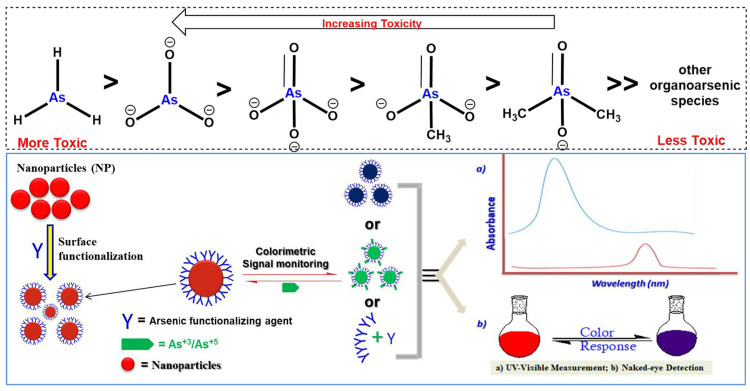
(**Top**) Toxicity pattern of arsenic compounds. (**Bottom**) General illustration of basic principle of an arsenic-based optical colorimetric nanosensor. Reprinted with permission from Ref. [23]; Copyright 2024 Springer.

By examining the recent advancements in nanomaterial-based colorimetric detection of arsenic in water, this review aims to provide researchers, policymakers, and environmental scientists with valuable insights into the potential solutions and innovations available for combatting arsenic contamination. Ultimately, the widespread adoption of these nanomaterial-based methods could contribute to a safer and healthier future, free from the threat of arsenic-contaminated water sources [22]. Figure 1 provides a visual explanation of the different toxicity levels of arsenic compounds, as well as the basic working principle of arsenic-detecting optical colorimetric nanosensors.

### Arsenic Contamination and Its Impact on Public Health

**Figure 2:**
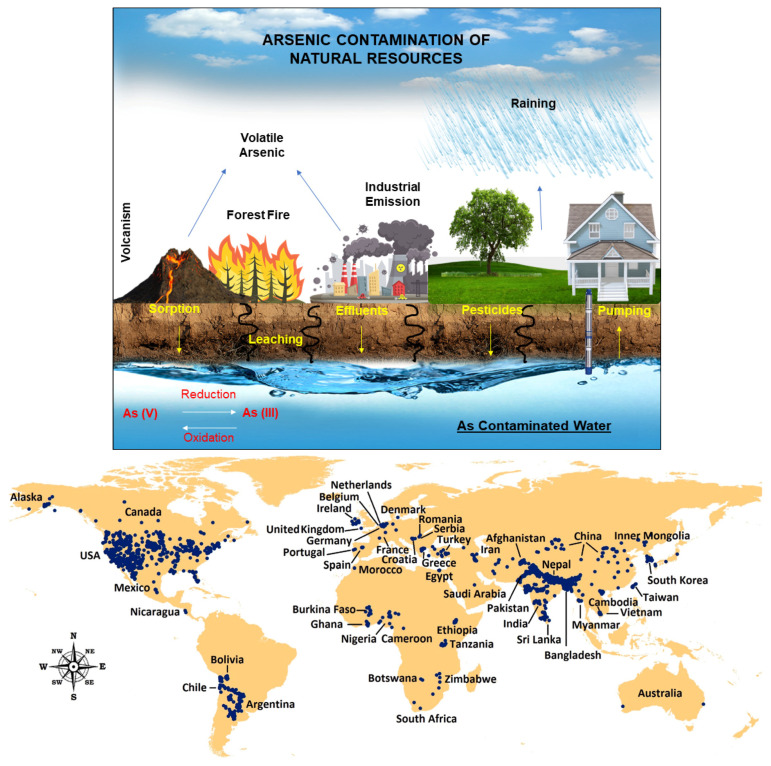
**Top**: Schematic representation of human exposure to arsenic and arsenic cycle. Reprinted with permission from Ref. [27]; Copyright 2023 The Royal Society of Chemistry. **Bottom:** Global occurrence of arsenic reported in major countries/regions with arsenic concentration ≥10 µg/L. Reprinted from Ref. [28], Copyright 2021 Elsevier.

Arsenic is a naturally occurring element that ubiquitously exists in both organic and inorganic form in the environment [24]. Arsenic contamination is a serious global health threat, particularly in countries like Bangladesh, Taiwan, India, Mexico, China, Chile, Argentina, and the USA. People are exposed to arsenic mainly through contaminated food and water, or by inhaling it during agricultural or mining work. [25,26]. Figure 2 illustrates how humans are exposed to arsenic and its cycle [27].

The World Health Organization (WHO) considers arsenic contamination a critical public health emergency. Their guidelines set a safe limit of 10 ppb of arsenic in drinking water, with an absolute maximum of 50 ppb [10,27,29]. 

Chronic arsenic poisoning is characterised by specific skin areas with pigmentation and keratosis. Pigmentation can also affect mucous membranes, such as the tongue under the surface or the buccal mucous membranes. Alongside malignancies, it can manifest in various dermatological conditions, such as hyperpigmentation, keratosis, and leuco-melanosis. Furthermore, arsenic exposure has been linked to cardiovascular diseases, respiratory ailments, neurological disorders, and reproductive issues. In addition, leuco-melanosis is common in arsenicosis patients. Numerous epidemiological studies have looked into the danger of different malignancies caused by arsenic absorption through drinking water [1,30,31,32]. The link between high arsenic levels in drinking water and cancer is undeniable. Studies show an increased risk of cancers in the skin, bladder, and lungs. This serious public health threat extends around the globe [33]. To identify and address contaminated water sources, accurately measuring arsenic concentrations in drinking water is crucial. The detection and purification of arsenic-contaminated water can help reduce the arsenicosis [34,35,36,37]. 

## 2. Colorimetric Sensing

Colorimetry and spectrophotometry, both techniques that analyse the interaction of light with matter to identify molecules, offer valuable tools for field applications. These methods achieve this by measuring the specific wavelengths of light a molecule absorbs or emits, creating a unique “spectral fingerprint” for identification.

Colorimetry, a simpler technique, focuses on measuring the absorbance of pre-defined colours, typically within the visible spectrum. This characteristic makes colorimetry well-suited for portable arsenic monitoring applications. Similar to traditional field test kits, colorimetric detection can be done visually by observing a colour change, or through digital imaging for more advanced analysis. The ease of use and availability of portable digital detectors like cameras, UV-Vis spectrometers modified for colorimetry, or even smartphones contribute to the field-friendliness of colorimetry.

Spectrophotometry, on the other hand, provides a more comprehensive analysis. It measures the transmittance or reflectance of light across a much broader range of wavelengths, not just pre-selected colours. In essence, colorimetry uses a limited set of fixed wavelengths within the visible range, while spectrophotometry offers a wider spectrum for analysis. This broader range of data allows spectrophotometers to achieve higher precision and greater versatility in colour analysis. They can assess the intricate details of spectral reflectance at various wavelengths, providing a more complete picture. However, this increased capability often comes at a higher cost compared to colorimeters [27].

### 2.1. Marsh Reaction and Gutzeit Method

The detection of arsenic in various samples, such as water, has been a critical area of analytical chemistry for over a century. Two historically significant methods developed in the 19th century, the Marsh Reaction and Gutzeit Method, remain important due to their foundational roles in arsenic analysis. Both methods leverage the generation of arsine gas (AsH_3_) as a central step, but they differ in their detection mechanisms and sensitivity, and they paved the way for more modern techniques like colorimetry.

The Marsh reaction, introduced by James Marsh in 1836, was a breakthrough in forensic toxicology. This method involves treating the sample with zinc and acid to produce arsine gas, which is then decomposed by passing it through a heated glass tube. The resulting metallic arsenic forms a characteristic black or brown mirror on the cooler parts of the tube, providing a sensitive and quantitative indication of arsenic presence. The Marsh reaction’s ability to detect minute quantities of arsenic (down to parts per billion) revolutionized the field, allowing for the precise identification of arsenic poisoning in forensic cases. Despite its complexity and the necessity for careful handling due to the toxic arsine gas, the method set a high standard for sensitivity in analytical chemistry [27,38]

In contrast, the Gutzeit method, developed by Heinrich Gutzeit in the late 19th century, offers a simpler, more accessible approach. This method also produces arsine gas from the reaction of the sample with zinc and acid, but it detects arsenic through a visible colour change. The arsine gas reacts with mercuric chloride on a filter paper, producing a yellow to brown stain indicative of arsenic. While not as sensitive or quantitative as the Marsh reaction, the Gutzeit method’s ease of use and minimal equipment requirements made it popular for field testing and preliminary screenings. However, its semi-quantitative nature and susceptibility to false positives from other substances limit its precision [17,39].

These methods laid the groundwork for modern techniques like colorimetry, which have further advanced arsenic detection. Colorimetric methods, such as the molybdenum blue colorimetric sensor, offer high sensitivity and specificity by relying on colour changes that can be quantitatively measured using spectrophotometers. These modern techniques combine the qualitative simplicity of the Gutzeit method with the quantitative accuracy of the Marsh reaction, providing robust and user-friendly solutions for environmental monitoring and public health. Thus, while the Marsh reaction and Gutzeit method were pivotal in their time, contemporary colorimetric methods build on their principles to achieve greater precision and applicability in arsenic detection. Table 1 summarizes the relevant literature on commercial kits based on the Gutzeit approach.

### 2.2. Molybdenum Blue-Based Methods

**Figure 3:**
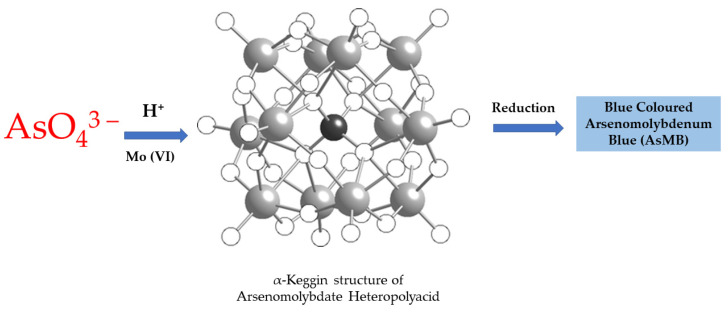
Molybdenum blue method for arsenate sensing and structure of arsenomolybdate heteropolyacid.

Similar to the Gutzeit method, the molybdenum blue assay is a colorimetric technique for arsenic detection. This assay relies on the specific reaction between arsenate ions and molybdate, forming a coloured heteropolyacid complex [45]. The procedural simplicity, involving primarily reagent mixing and passive flows, lends itself well to implementation within microfluidic devices. However, a major challenge is the potential interference from naturally occurring phosphates and silicates, which compete for reaction with molybdate. Therefore, accurate determination necessitates their removal from the sample solution prior to analysis [46,47]. The underlying principle of the assay involves the initial formation of an α-Keggin arsenomolybdate heteropolyacid from the reaction of arsenate (As(V)) with molybdate. Subsequent reduction of this complex yields the characteristic heteropoly blue compound, enabling colorimetric quantification of arsenic. Importantly, arsenite (As(III)) does not directly form this complex, requiring pre-oxidation to As(V) for accurate detection by this method [48]. The mechanism and structure for the same is shown in the reaction below and Figure 3.

As(V) + Mo(IV) → [AsMo_12_O_40_]^3−^ → [AsMo_12_O_40_]^7−^

Research has been concentrated on addressing the interference from other ions commonly present in environmental samples, ensuring the specificity of the molybdenum blue method. Strategies involving selective preconcentration or sample preparation techniques have been explored to improve accuracy and reliability in complex sample matrices. 

The future trajectory of the molybdenum blue colorimetric sensor for arsenic detection is poised for significant advancements. Innovations will focus on the integration of advanced nanomaterials, which promise to enhance the sensor’s sensitivity and detection capabilities. Concurrently, efforts in miniaturization aim to create more compact, portable devices suitable for on-site arsenic monitoring. Additionally, improvements in selectivity are critical, ensuring that these sensors can accurately detect arsenic even in the presence of other potentially interfering substances. These developments collectively strive to meet the growing demand for reliable, field-deployable tools, essential for maintaining water safety and protecting environmental health.

### 2.3. Methylene Blue-Based Methods

An alternative colorimetric technique for arsenic detection leverages the direct interaction between arsenic and the cationic dye, methylene blue. This approach relies on the reduction of methylene blue by arsine gas, leading to a loss of its characteristic coloration. To accelerate this reaction, anionic micelles are employed as catalysts, while silver nanoparticles act as electron transfer mediators between arsine gas and the dye [49]. Unlike standard field kits, the arsine gas produced by this method remains in solution during the examination, lowering the technician’s risk of exposure. Microfluidic adaptations for both of these technologies can also be created. 

Modern field test kits offer significant improvements over traditional methods [50]. Digital readouts provide precise arsenic concentration measurements instead of estimations. Digital colour processing eliminates human error associated with visual colour matching charts. Examples of these advancements include the Arsenator by Wagtech and the DigiPAsS by Palintest. Additionally, modern kits incorporate components to remove interfering gases like hydrogen sulphide, enhancing accuracy. A study comparing eight commercial kits in Bangladesh with a highly accurate lab technique (HG-AAS) revealed a correlation between price and performance. The most expensive kits (LaMotte and Quick II) offered the most accurate measurements, while cheaper kits showed lower precision and accuracy [27].

## 3. Nanomaterials and Colorimetric Advancements

Many researchers have developed advanced detecting systems by using the unique properties of nanoparticles, such as their large surface area and adjustable surface capabilities. When exposed to arsenic ions, functionalized nanoparticles, such as gold nanoparticles and quantum dots, exhibit unique changes in their optical characteristics, allowing for quick and exact detection. These nanoparticles not only improve sensitivity by selectively attaching to arsenic, but they also allow for straightforward visual identification and quantification using basic optical equipment. A significant advancement in arsenic sensing technology has been made with the incorporation of nanomaterials into colorimetric arsenic detection methods. This shows a promising path towards the creation of portable, highly sensitive, precise, and dependable on-site monitoring tools for a variety of environmental and industrial applications [51,52,53,54].

### 3.1. Carbon Nanomaterials

**Figure 4:**
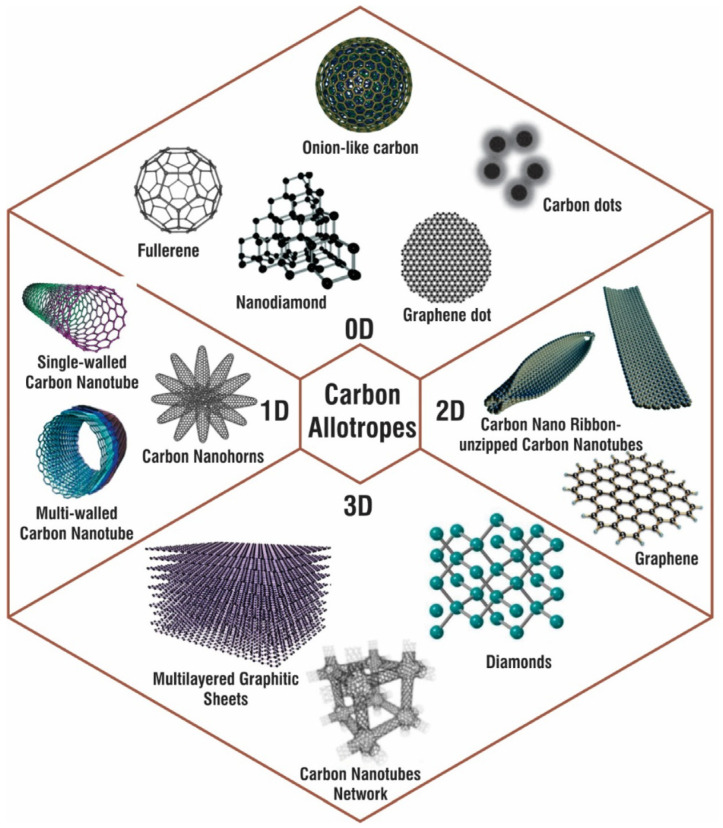
Various nanoforms of carbon allotropes with examples for 0D, 1D, 2D, and 3D carbon nanostructures. Reproduced under the terms of the CC-BY license from Ref. [61]; Copyright 2021 The Authors, published by MDPI.

Carbon is one of the most abundant elements on Earth, occurring naturally as graphite, diamond, and coal in its fundamental forms. Various nanoforms of carbon allotropes with examples for 0D, 1D, 2D, and 3D carbon nanostructures are shown in Figure 4. Its nanostructured allotropes have been extensively researched over the past twenty years due to their unique hybridization properties and their responsiveness to changes during synthesis, enabling precise adjustments to their material properties. Carbon can exist in various hybridization states, which significantly influence its chemical, mechanical, thermal, and electrical properties. This versatility allows carbon materials to be tailored for a wide range of applications. Various carbon-based materials have been explored in numerous sensing applications [55,56,57,58,59,60,61]. This adaptability is particularly relevant in the context of arsenic detection, where the specific properties of nanostructured carbon allotropes can be leveraged for enhanced sensitivity and selectivity.

It is also explored in arsenic detection, demonstrating their potential for developing sensitive and selective detection systems. Through the manipulation of carbon nanotubes (CNTs), graphene, carbon quantum dots, and carbon nanofibers, researchers have been able to harness their unique properties to create tailored sensing platforms. Functionalization of these carbon-based nanomaterials with specific recognition elements, such as aptamers, antibodies, or chemical ligands, enables selective binding to arsenic ions present in environmental samples. The binding interaction induces measurable changes in the electrical conductivity, optical properties, or fluorescence emission of the nanomaterials, which can be exploited for sensing purposes. Furthermore, the high surface area, excellent chemical stability, and tuneable surface chemistry of carbon-based nanomaterials contribute to their sensitivity, preciseness, selectivity, and rapid response, making them highly desirable for addressing the challenges of arsenic contamination in various settings. Continued research and development in this field hold significant promise for advancing arsenic detection technologies and mitigating the associated health and environmental risks. 

**Figure 5:**
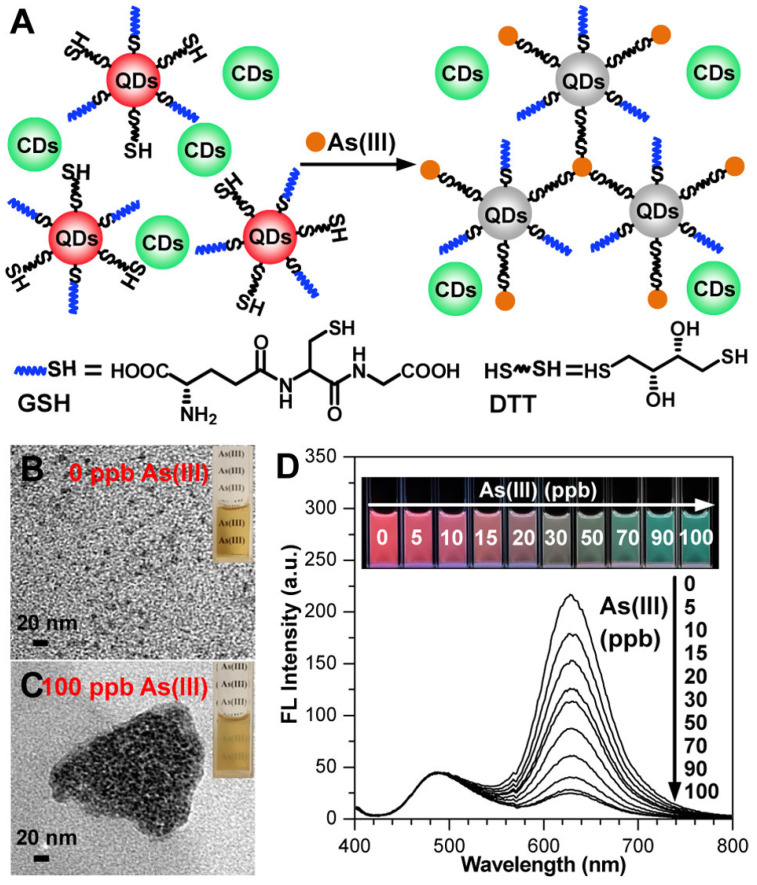
(**A**) Visualization mechanism of As(III) using GSH/DTT-QDs as fluorescent sensory probe and CDs as an internal standard probe. (**B**,**C**) TEM images of GSH/DTT-QDs before and after the addition of As(III) (insets are corresponding optical photos under daylight). (**D**) Fluorescent spectra of mixing GSH/DTT-QDs/CDs (20/10 μL in 1.5 mL of Tris-HCl buffer, pH 7.4) with addition of As(III). Inset photos show the evolution of corresponding colours under 365 nm UV lamp. Reprinted with permission from Ref. [63]; Copyright 2016 American Chemical Society.

A study by He et al. developed carbon dot (CD)–MnO_2_ nanocomposites for detecting As(III) with high sensitivity and selectivity. The intense blue fluorescence of CDs, quenched by MnO_2_ via the FRET effect, is restored upon reaction with As(III), allowing detection down to 1.40 ppb [62]. Another research presents a fluorescent test paper that visually detects As(III) levels as low as 5 ppb through colour variations, offering a promising tool for environmental and food assays (Figure 5) [63]. 

A recent study unveiled an innovative sensor design that combines high sensitivity, selectivity, and affordability. This novel sensor utilizes surface plasmon resonance (SPR) technology integrated with a nanocomposite comprising hydrous ferric oxide, magnetite, and reduced graphene oxide. This composite structure enhances the sensor’s ability to detect arsenic ions with remarkable precision, achieving an impressive detection limit of 0.1 parts per billion (ppb). By leveraging the unique properties of these materials and the prism-based SPR configuration, the sensor offers a potent solution for accurately identifying arsenic contamination in various environmental samples [64]. A new sensor based on magnetic graphene quantum dots offers advanced sensitivity and selectivity for detecting arsenic contamination. The sensor based on magnetic graphene quantum dots demonstrated superior performance compared to sensors utilizing zinc oxide (ZnO) and cadmium sulphide (CdS) quantum dots. The enhanced performance of the magnetic graphene quantum dot sensor can be attributed to the presence of iron oxide, which facilitates greater interaction sites for the formation of chelating complexes with arsenic in the testing medium. This heightened interaction capability allows for more efficient and selective detection of arsenic ions, showcasing the potential of magnetic graphene quantum dot-based sensors in environmental monitoring and analysis applications [65]. A research group utilized L-cysteine functionalized graphene quantum dots (GQDs) for optical detection of As(III) with remarkable precision and selectivity, achieving detection within sub-ppb concentrations. The study detailed in the research paper demonstrated that the optical response of GQDs rapidly changed upon contact with As(III) across a concentration range from 0.025 to 25 ppb, encompassing the permissible limit of arsenic in drinking water. These findings highlight the excellent sensitivity and selectivity of L-cysteine functionalized GQDs for detecting As(III), showcasing their potential for environmental monitoring applications [66].

### 3.2. Metallic Nanoparticles (MNPs)

#### 3.2.1. Gold-Based Nanomaterials (AuNPs)

Gold nanoparticles (AuNPs) have emerged as a prominent tool in colorimetric arsenic detection owing to their unique optical properties and surface chemistry. Functionalized AuNPs can be tailored to selectively bind with arsenic ions, leading to distinct changes in their optical characteristics, which are easily observable by the naked eye or through simple optical instruments [67,68,69].

In arsenic detection, the interaction between AuNPs and arsenic ions induces aggregation or dispersion of the nanoparticles, resulting in a shift in their SPR band. This change in SPR leads to a visible colour alteration, serving as a reliable indicator for the presence and concentration of arsenic. Additionally, the size, shape, and surface modifications of AuNPs can be precisely engineered to enhance their precision and selectivity towards arsenic, enabling improved detection limits and minimizing interference from other ions present in environmental samples.

Despite their promising attributes, challenges such as ensuring specificity in complex sample matrices and refining detection limits persist. Researchers continue to explore novel approaches involving AuNPs to develop more robust and sensitive colorimetric arsenic detection methods suitable for practical applications in diverse environmental settings. Efforts in optimizing AuNP-based detection systems aim to address these challenges and facilitate their broader utilization for efficient and reliable arsenic monitoring.

**Figure 6:**
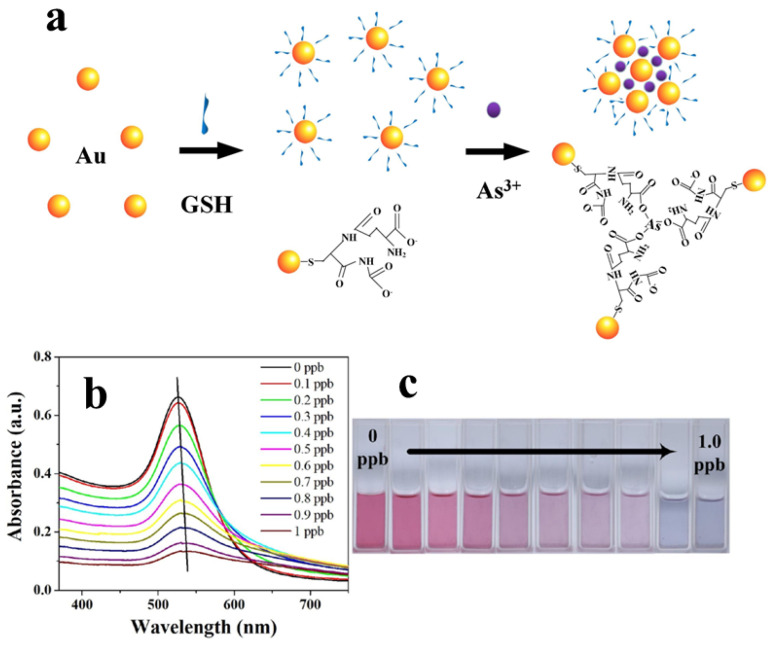
(**a**) Schematic illustration of the mechanism for the detection of arsenic by GSH functionalized AuNPs. (**b**,**c**) Absorption spectra and photographs of GSH-AuNPs after addition of different concentrations of arsenic (0.1–1 ppb). Reprinted with permission from Ref. [70]; Copyright 2021 Elsevier.

Figure 6a provides a schematic representation of the colorimetric detection of arsenic ions using a GSH-AuNPs solution. The illustration depicts the formation of As-O bonds between arsenic ions and functionalized ligands present on the AuNPs. This bonding event induces pronounced aggregation of the AuNPs, resulting in a swift and visually discernible colour change. Figure 6b,c illustrates the absorption spectra of GSH-AuNPs solution after the addition of different concentrations of arsenic ions and also shows the corresponding photographs of GSH-AuNPs solutions [70]. The sensitivity of this method allows for the detection of arsenic ions down to 0.12 ppb.

**Figure 7:**
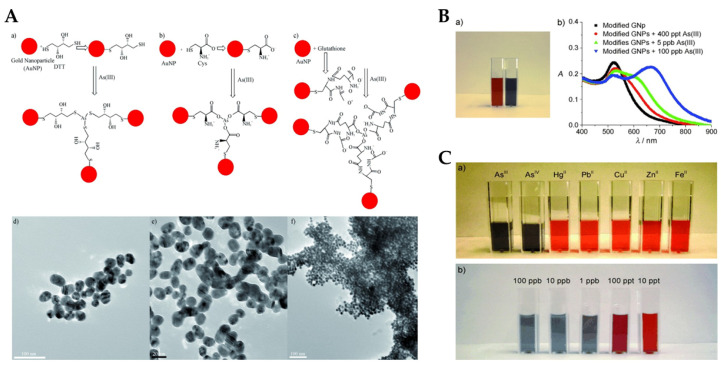
(**A**): Representation of AuNP-based arsenic detection. (**a**) DTT-modified AuNPs; (**b**) Cys-modified AuNPs; (**c**) GSH-modified AuNPs; (**d**) TEM image showing GSH/DTT/Cys-modified AuNPs before the addition of arsenic (III); (**e**) TEM image demonstrating aggregation of GSH/DTT/Cys-modified AuNPs after addition of 80 ppb arsenic (III); (**f**) TEM image after the addition of 250 ppt arsenic (III). (**B**): (**a**) Photograph showing colorimetric change of GSH/DTT/Cys-modified gold nanoparticles upon addition of 800 ppb arsenic (III); (**b**) absorption profiles of modified gold nanoparticles before and after addition of arsenic (III) ions. (**C**): (**a**) photograph showing colorimetric changes of GSH/DTT/Cys-modified gold nanoparticles in the presence of PDCA upon addition of various metal ions (5 ppb) and (**b**) different concentrations of arsenic (III). Reprinted with permission from Ref. [72]; Copyright 2009 Wiley.

**Figure 8:**
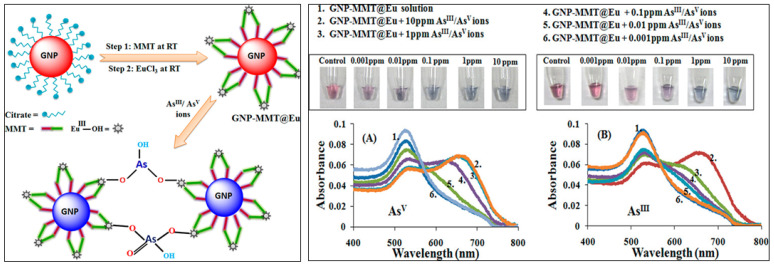
**Left:** Schematic of the fabrication of the gold nanosensor, GNP-MMT@Eu, and its interaction with arsenic ions (As^III^/As^V^) in water. **Right:** UV–vis spectra of GNP-MMT@Eu in the presence of increasing concentrations of (**A**) As^V^ and (**B**) As^III^ ions showing gradual decrease in absorbance. Inset: image of various concentrations of As^V^ (**A**) and As^III^ (**B**) solutions treated with GNP-MMT@Eu showing gradual change in colour from red to blue due to aggregation of the gold nanosensor. Reprinted with permission from Ref. [74]; Copyright 2018 American Chemical Society.

**Figure 9:**
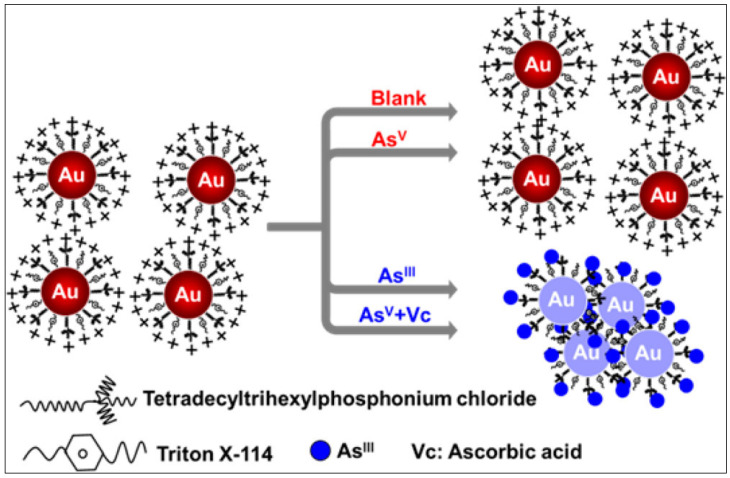
Schematic representation of the mechanism of P-AuNPs for determination of As^III^ and As^V^. Reprinted with permission from Ref. [75]; Copyright 2014 American Chemical Society.

Surface modification of AuNPs with sulphur-containing ligands significantly improves the sensitivity and preciseness of colorimetric arsenic sensors. This enhancement stems from arsenic’s strong binding affinity towards sulphur moieties. Functionalization with glutathione (GSH), dithiothreitol (DTT), cysteine (Cys), and 2,6-pyridine dicarboxylic acid (PDCA) has proven effective in detecting As(III) in aqueous environments [71]. DTT-conjugated AuNPs demonstrate particular efficacy due to their accessible SH groups, facilitating direct As-S bond formation (Figure 7A). GSH and Cys, lacking readily available SH groups, exhibit a different interaction mechanism. The colorimetric response of these modified AuNPs to As(III) is visualized in Figure 7B. Incorporation of PDCA offers superior selectivity for As(III). Unlike the SH-mediated interaction of DTT, GSH, and Cys, PDCA’s unique binding mode (Figure 7C) minimizes interference from other species [72]. This method offers an excellent detection limit (10 ppt) and exhibits strong selectivity over other analytes. Deepa et al. presented a highly efficient colorimetric sensor based on 2,4-dinitrophenyl hydrazones for the selective and sensitive detection of arsenite ion in aqueous medium [73]. This sensor provides a remarkably low detection limit of 0.35 × 10^−6^ M for As(III) in aqueous medium, delivering a cost-effective, colorimetric solution with superior precision compared to other reported methods. In another study, Nath et al. demonstrated a europium-functionalized gold nanosensor (GNP-MMT@Eu) for arsenic detection, highlighting its visible colour change in the presence of As(III) and As(V) ions (Figure 8) [74]. In another study, the mechanism of P-AuNPs in the determination of As(III) and As(V) was schematically represented by Tan et al. (Figure 9). The limit of detection (LOD) for both As(III) and Arsenic(V) was determined to be 7.5 ppb [75].

#### 3.2.2. Silver-Based Nanomaterials

Silver nanoparticles (AgNPs) have gained attention in the realm of colorimetric arsenic detection due to their unique properties, including their optical characteristics and SPR. These nanoparticles exhibit a distinct colour change in the presence of arsenic ions, offering a simple and visually detectable signal [76]. Additionally, AgNPs hold promise due to their tuneable sizes, which can influence the sensitivity and selectivity of arsenic detection. By modifying the size and surface chemistry of AgNPs, researchers can fine-tune their interactions with arsenic ions, enhancing the specificity and sensitivity of the colorimetric assay. 

**Figure 10:**
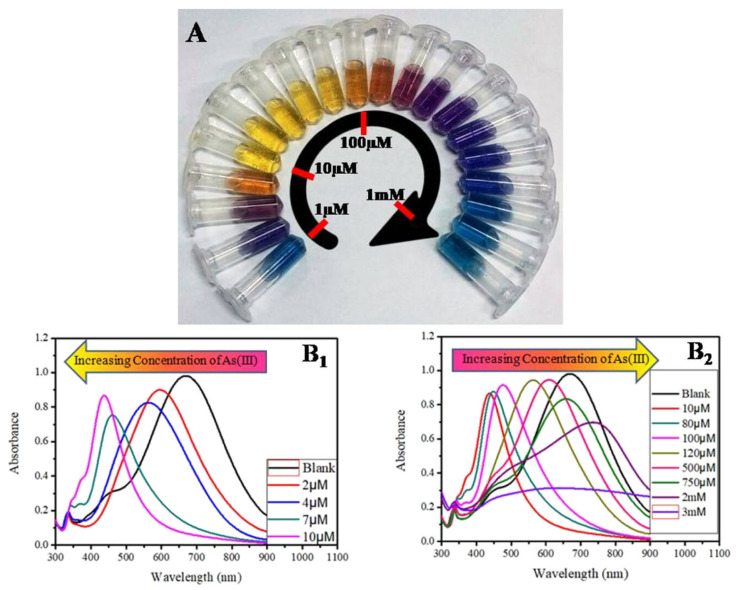
(**A**) Concentration-dependent colour-coded sensing of arsenic (III) between the concentration range of 10^−6^ to 10^−3^ M. (**B_1_**,**B_2_**) Tuning of SPR as a result of morphological change of AgNPr (silver nanoprism) at different concentrations of arsenic (III) between 10^−6^ and 10^−3^ M, where (**B_1_**) shows the variation of plasmon band at different lower concentrations of arsenic (III) in the range of 0.0–10.0 μM (0.0 μM (blank): black trace (λ_max_ = 704 nm), 1.0–2.0 μM: blue trace, 2.0–4.0 μM: orange trace, 5.0–7.0 μM: red-violet trace, 8.0–10.0 μM: blue-violet trace) and (**B_2_**) at different higher concentrations of arsenic (III). The plasmon band, and hence the colour of the nanomaterials, changes in a distinct manner, where a specific colour remains unchanged in a broader range of growing concentrations such as: 10.0–80.0 μM: yellow, 90.0–100.0 μM: orange, 110.0–200.0 μM: dark red, 250.0–500.0 μM: purple, 750.0 μM to 2 mM: different shades of blue, 3–10 mM: faded blue, and above 10 mM the colour becomes faint blue to grey or almost colourless. Reprinted with permission from Ref. [78]; Copyright 2019 American Chemical Society.

A study by Boruah et al. demonstrated the effectiveness of polyethylene glycol (PEG)-functionalized AgNPs for detecting As(III) ions in water at concentrations as low as 1 ppb. The addition of PEG provides two key advantages. First, it grants the nanoparticles adjustable negative charges, promoting stability and preventing clumping (agglomeration). More importantly, PEG’s hydroxyl groups interact with As(III) ions, causing the initially yellow AgNPs to aggregate and turn bluish. This colour change serves as a simple detection method for arsenic contamination [77]. Another research effort described the development of an assay using silver nanoprisms (AgNPrs) for As(III) detection. This method leverages the unique ability of AgNPrs to modify their SPR (tuning-detuning) in response to specific molecules (Figure 10). This approach not only confirms the presence of As(III) but also allows for concentration detection up to 75 ppb. The authors highlight the assay’s potential for field applications due to its cost-effectiveness and ability to analyse real water samples [78]. 

**Figure 11:**
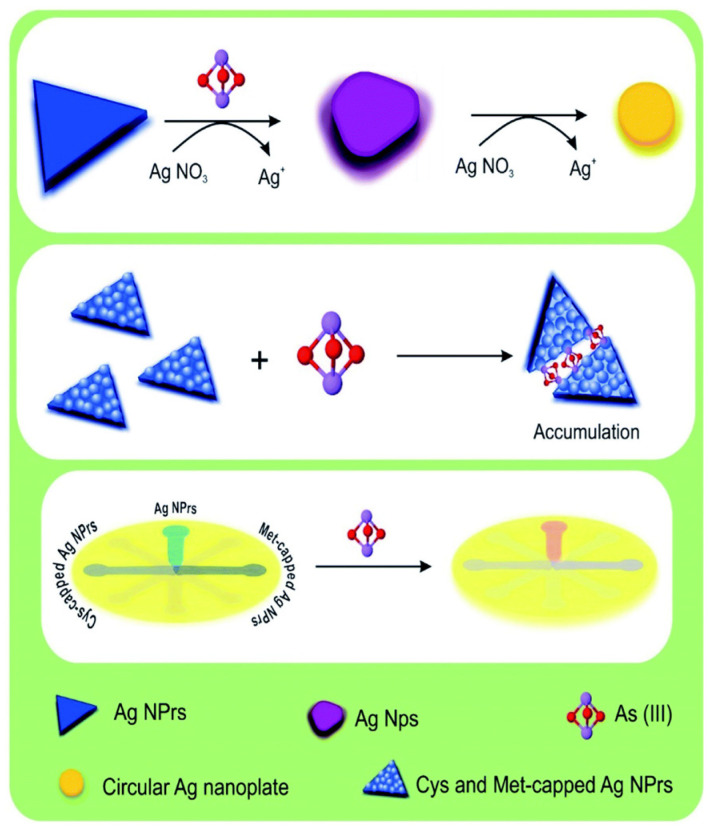
Schematic illustration of naked-eye detection of As(III) by colorimetric assay based on AgNPs. Reprinted with permission from Ref. [79]; Copyright 2022 The Royal Society of Chemistry.

A rapid and sensitive colorimetric method employing silver nanoprisms (AgNPrs) has been developed for the accurate detection of arsenic in water and human urine (Figure 11) [79]. The method relies on a redox reaction between silver nitrate and As(III), which induces a morphological transformation in the AgNPrs. This transformation results in a discernible colour change from blue to purple, providing a visual indicator of arsenic presence. UV-Vis absorption spectra confirm the morphological change. The method demonstrates a linear relationship between the change in adsorption peak and arsenic concentration (0.0005 to 1 ppm), with a lower limit of quantification (LLOQ) of 0.0005 ppm [79].

### 3.3. Metal Oxide-Based Nanocomposites

Metal oxide-based nanoparticles have emerged as promising candidates for colorimetric arsenic detection, offering distinct advantages in sensitivity and specificity owing to their unique properties. Metal oxide nanoparticles, such as iron oxide (Fe_2_O_3_), titanium dioxide (TiO_2_), and zinc oxide (ZnO), possess excellent surface reactivity and customizable surface functionalities. These nanoparticles can be modified to selectively interact with arsenic ions, inducing alterations in their optical properties or surface chemistry. These changes manifest as visible colour shifts, providing a basis for colorimetric detection. Moreover, the versatility of metal oxide nanoparticles allows their incorporation into composite materials, enhancing stability and sensitivity, particularly in complex environmental samples where interference from other ions may occur.

Metal oxide nanoparticles exhibit tuneable properties, making them attractive for tailoring sensitivity, selectivity, and stability in colorimetric sensing of arsenic. Ongoing research efforts aim to optimize nanoparticle design, surface modifications, and hybrid material development to overcome challenges and further their application in robust and reliable colorimetric arsenic detection methodologies.

In a study by Christus et al., a novel colorimetric sensor was developed for the detection of arsenate in aqueous solution using Fe_3_O_4_ nanoparticles. The Fenton-like catalytic reaction of Fe_3_O_4_ NPs promoted the oxidation of methylene blue indicator in the presence of H_2_O_2_, resulting in a change in colour. The proposed colorimetric sensor exhibited rapid and selective detection of As(V) in aqueous solution with a detection limit of 0.358 nM [80]. 

### 3.4. Metal–Organic Frameworks (MOFs) and Covalent Organic Frameworks (COFs)

Metal–organic frameworks (MOFs) and covalent organic frameworks (COFs) are advanced porous materials with incredible potential for various applications. MOFs are built like 3D tinker toys, with metal clusters acting as joints and organic molecules as the connecting rods. This structure creates a vast network of tiny pores. COFs work similarly but are made entirely of organic building blocks linked together by strong covalent bonds. Both MOFs and COFs can be designed with specific pore sizes and chemical properties, making them useful for trapping gases, filtering substances, delivering drugs, and acting as catalysts. [81,82]. MOFs and COFs have garnered attention as promising platforms for arsenic colorimetric detection. Their exceptionally high surface area, adjustable internal structures (pore topologies), and ability to be customized with specific functional groups offer remarkable advantages. MOFs, with their combination of metal ions/clusters and organic linkers, and COFs, with their exclusively organic building blocks, provide diverse and adaptable frameworks for designing sensors that react to arsenic with visible colour changes. MOFs can be generated to have particular binding sites that selectively capture arsenic ions from a sample in arsenic detection. MOFs that have been functionalized with appropriate organic linkers or functional groups can recognise and bind arsenic. This interaction causes changes in the optical characteristics of the MOF, resulting in noticeable colour changes that can be observed with the naked eye or basic optical tools.

Furthermore, the immobilisation of other indicators or chromophores within the structures of MOFs is made possible by their vast surface area, which improves the sensitivity and specificity of arsenic detection. Researchers can optimise the MOF’s performance for effective and precise colorimetric detection of arsenic in various environmental samples by fine-tuning its composition and pore properties. To combat arsenic contamination, researchers are exploring the use of MOFs for colorimetric detection. Recent studies show that MOFs and their composites offer the potential for extremely sensitive, selective, and easily transportable sensing platforms.

**Figure 12:**
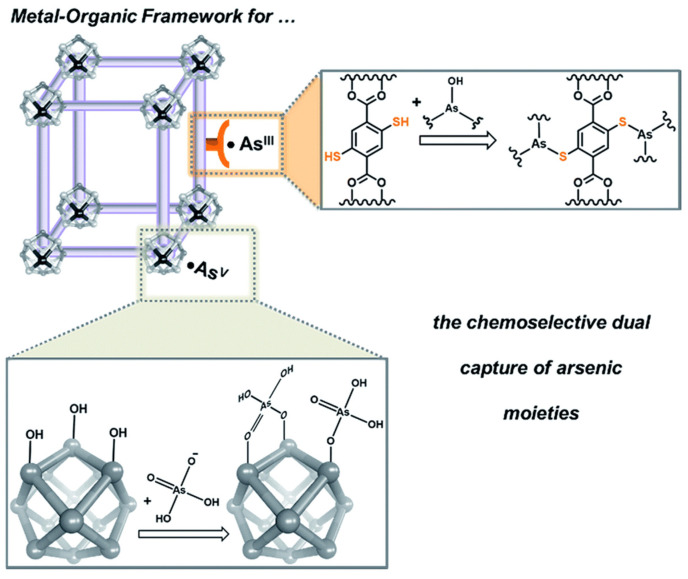
A schematic representation that suggests how MOFs can be tailored to coordinate anionic As^V^ moieties at the node while binding neutral As^III^ with the linkers. Reprinted with permission from Ref. [83]; Copyright 2016 The Royal Society of Chemistry.

**Figure 13:**
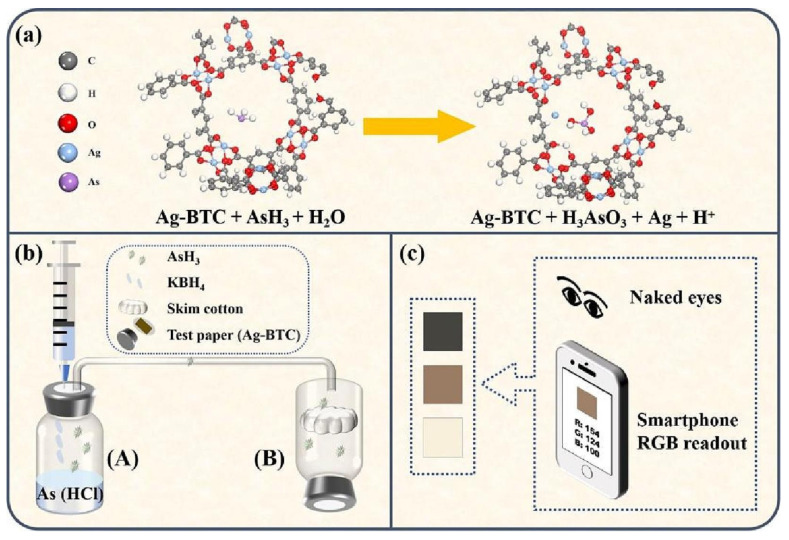
The schematic diagram for the smartphone-based colorimetric system. (**a**) The reaction of Ag-BTC and AsH_3_. (**b**) The schematic diagram of the hydride generation–colorimetric system. (**c**) Readout by naked-eye and smartphone RGB mode simultaneously. Reprinted with permission from Ref. [84]; Copyright 2023 Elsevier.

Figure 12 depicts a MOF modification used to coordinate As(V) moieties at the node. Amino-decorated MOF compounds are fascinating. The amino-functionalized iron-based MOFs demonstrated high selectivity for As(III) detection [83]. When exposed to water, the most often seen MOF structure bent. To increase the water stability of ligand-based carboxylate MOFs, high-valence metal ions such as Cr(III), Zr(IV), Fe(III), and Al(III) were utilized to form chemically stable coordination bonds. The addition of ligands with hydrophobic activity, such as methyl, ethyl, and trifluoromethyl, is critical for protecting metal bodies against hydrolysis. Zou et al. presented a schematic for a smartphone-based colorimetric system designed for arsenic detection, including its hydride generation component (Figure 13). Visual colorimetric and smartphone RGB readout modes were used for As(III) detection, with a detectable limit of 10 ppb for smartphone and 50 ppb for naked-eye, respectively [84].

**Figure 14:**
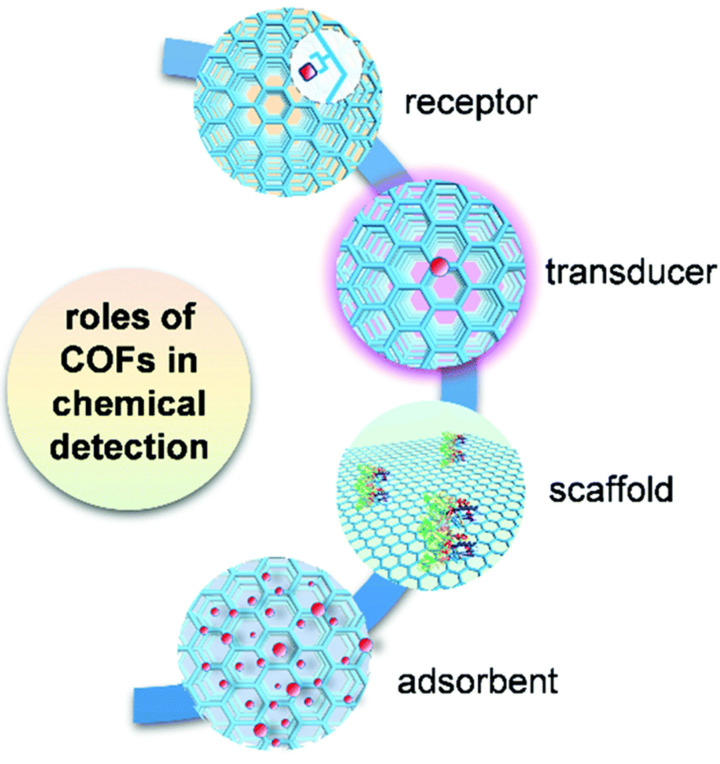
Multiple roles of COFs in the application of chemical detection. Reprinted with permission from Ref. [85]; Copyright 2021 The Royal Society of Chemistry.

COFs hold considerable promise as materials for arsenic colorimetric detection. Their high surface area, tuneable pore sizes, and chemical stability make them attractive candidates for this application. By incorporating arsenic-binding ligands into the structure of COFs, researchers can create selective arsenic capture systems. Additionally, the porous nature of COFs allows for rapid diffusion of analytes, enhancing detection sensitivity. When combined with colorimetric indicators or nanoparticles, COFs can facilitate visible colour changes upon arsenic binding, enabling straightforward and rapid detection without the need for complex instrumentation. Given these characteristics, COFs present themselves as a promising avenue for the development of arsenic detection methods, offering versatility and effectiveness in addressing arsenic contamination challenges. Figure 14 illustrates the diverse functions of COFs in various chemical detection applications [85].

Yin and Liu reported a fluorescence-based “turn-on” mode sensor for As(III) detection using a functional COF constructed from bipyridine (Dpy-TFPB). The Dpy-TFPB framework employs its nitrogen-rich sites as selective receptors for As(III) ions, while the material’s π-conjugated system acts as the signal transducer. As(III) binding disrupts the photoinduced electron transfer (PET) process within Dpy-TFPB, leading to a significant increase in fluorescence intensity. This approach exhibited high sensitivity with an ultra-low detection limit of 8.86 nM for As(III) [86]. In another study, Chen et al. employed COFs for the first time to simultaneously detect and adsorb organic arsenic from water [81]. They synthesized two highly porous, isoreticular, sp^2^ carbon-conjugated COFs and subsequently functionalized them with amidoxime groups through post-synthetic modification (PSM).

### 3.5. Other Materials and Combinations

In the colorimetric detection of arsenic, integration with enzymes, aptamers, and antibodies can significantly enhance sensitivity and specificity. Enzymes like arsenate reductase or arsenite oxidase can catalyse reactions, leading to the generation of colorimetric signals upon exposure to arsenic. Aptamers, single-stranded DNA or RNA molecules selected to bind specifically to arsenic, can be utilized as recognition elements. Upon binding to arsenic, aptamers undergo conformational changes that can be transduced into colorimetric signals through various means such as the displacement of complementary DNA strands linked to AuNPs. Similarly, antibodies specific to arsenic can be employed for recognition, where upon binding to arsenic, the antibodies can initiate a colorimetric response, such as aggregation-induced colour change of AuNPs linked to antibodies. However, incorporating these biomolecules presents challenges; enzymes may suffer from stability issues, aptamers can be prone to degradation, and antibodies may exhibit cross-reactivity. Additionally, careful optimization of immobilization strategies onto detection platforms is necessary to maintain their functionality. Addressing these challenges through innovative engineering and optimization approaches is crucial to harnessing the full potential of enzymes, aptamers, and antibodies in enhancing the colorimetric detection of arsenic. Table 2 summarizes some examples of work on nanomaterials advancement in colorimetric detection of arsenic.

## 4. Challenges and Future Prospects

Despite the recent advancements and developments, there are still many challenges that need attention and require interdisciplinary collaboration between scientists, engineers, and technologists to develop robust, cost-effective, and reliable nanomaterial-based systems for arsenic detection in water to ensure safe drinking water for all [32,113,114,115].

### 4.1. Challenges

- Interferences and Matrix Effects: Despite the sensitivity exhibited by certain nanomaterials-based systems, their susceptibility to interference from other metal ions commonly present in groundwater poses a significant challenge. Ensuring the specificity of nanomaterial-based detection systems towards arsenic ions is crucial to avoid false positives or interference from other ions in water. This could be very crucial due to the complex environmental sample matrices in real-world analysis and can significantly influence the performance. Functionalization of nanomaterials can impart selectivity towards arsenic and minimize interference from other contaminants in water samples. Also, addressing matrix effects through sample pre-treatment techniques or matrix-matched calibration standards is crucial for reliable arsenic detection in diverse environmental analysis.
- Stability and Reproducibility: Maintaining the stability of nanomaterials and ensuring reproducibility of results are critical challenges that need to be addressed and validated to make these detection systems reliable for long-term use and practical application. Results variation is very common among nanomaterial-based methods/systems and needs to be further explored and validated with statistically significant datasets including the consistency of nanomaterials, integration with other sensor/system components, and the capability to adapt to broader sample types.
- Cost and Integration with Existing Infrastructure: While nanomaterial-based detection systems hold promise for arsenic detection, ensuring their cost-effectiveness and portability remains imperative. The affordability of nano-systems is crucial for widespread adoption, especially in resource-limited settings. Additionally, enhancing portability can enable on-site testing, facilitating timely interventions to ensure water safety. Scaling up the production of nanomaterials for large-scale deployment in arsenic detection systems while maintaining quality control and safety are challenges which need to be addressed. Integration of nanomaterial-based detection systems with existing water testing and treatment infrastructure and regulatory frameworks may pose logistical challenges and require collaborative efforts between researchers, policymakers, and stakeholders.
- Environmental Impact: The environmental impact of nanomaterials used in detection systems needs to be thoroughly evaluated to prevent any adverse effects on ecosystems and human health.

### 4.2. Prospects

- Nanomaterials have exhibited immense potential in the field of colorimetric detection of arsenic in water due to their unique properties such as high surface area, tuneable optical properties, and high sensitivities. They are rapid, cost-effective and can offer high precision towards arsenic detection due to their large surface area to volume ratio, enabling the detection of low As concentrations. Nanomaterial-based detection systems can be designed to be portable and user-friendly, enabling their deployment in remote or resource-limited areas where arsenic contamination is prevalent.
- Future research should emphasize the creation of cost-effective, user-friendly, greener, and environmentally sustainable nanoparticles/nanomaterials. Prioritizing selectivity, sensitivity, low detection limits, anti-interference capabilities, and simplicity in design will ensure widespread accessibility and application of arsenic detection technologies. Focus on reliable and consistent synthetic methods yielding nanoparticles of regular size is crucial, as nanoparticle size plays a key role in As(III) detection to improve their practical application.
- Advanced materials such as carbon dots (CDs), MXene, graphene, and MOFs/COFs offer distinct features that hold promising potential for advancing arsenic colorimetric detection. CDs are capable of exhibiting high sensitivity due to their tuneable properties and easy surface functionalization for selective arsenic binding [116]. MXene materials boast high surface areas and conductivity, which could facilitate efficient arsenic adsorption and detection. Graphene’s large surface-to-volume ratio, conductivity, and chemical tunability are of interest, while MOFs/COFs are capable of providing tailorable pore structures for precise arsenic sorption and integration with signal transduction mechanisms.
- Paper-based sensors could offer a promising solution for arsenic detection in water due to their simplicity, low cost, portability, and potential integration with existing infrastructure. These sensors involve immobilizing selective receptors or indicators onto paper substrates, undergoing a colorimetric change in the presence of arsenic ions. Further improvements could enhance specificity and sensitivity, and integration with smartphone technologies could enable real-time monitoring and remote sensing. Paper-based sensors hold great promise especially in resource-limited or remote areas where conventional methods are impractical.
- Microfluidic technology could offer innovative solutions for arsenic detection in water, leveraging miniaturized fluid-handling systems. Advantages include miniaturization, automation, precise fluid flow control, and integration of multiple functions on a single device. Microfluidic platforms efficiently handle small sample volumes, reducing reagent consumption and waste. It can help in multiple samples analysis simultaneously to enhance monitoring capabilities.

Addressing these challenges and exploring the prospects above would enable the development of robust, sensitive, and selective arsenic detection platforms crucial for ensuring water safety and environmental health. Artificial intelligence (AI) and the Internet of Things (IOT), including appropriate databases, could help in making portable, rapid, and compact devices to advance multiple analysis and on-site monitoring aspects [93].

## 5. Conclusions

This review has covered colorimetric approaches and nanomaterials advancements and role in colorimetric arsenic detection. The current challenges and future perspectives to advance the colorimetric arsenic sensing are discussed. Several nanomaterials have been discussed to contribute to the realization of sensitive, selective, and reproducible techniques for arsenic detection. Advanced materials and nanomaterials can improve the overall sensing properties as well as generate detectable signals to detect arsenic directly or indirectly. The discussion explored the current status and ongoing efforts to enhance signal and analytical performances, as well as the nanomaterials advancements which could boost and advance colorimetric arsenic detection towards combatting this global health concern. Addressing the above challenges and beyond by exploring these prospects would enable the development of robust, sensitive, and reliable arsenic detection platforms crucial for ensuring water safety and environmental health. The utilization of novel materials, biomaterials, and advanced functional nanomaterials could be crucial to develop cost-effective and robust detection systems. Integration with the IoT could further help in making portable, rapid, and compact devices capable of on-site and real-time monitoring.

## Figures and Tables

**Table 1:** Comparison of arsenic kit based on Gutzeit method. Reprinted with permission from Ref. [27]; Copyright 2023 The Royal Society of Chemistry.

Commercial Colorimetric Kit	Theoretical LOD (ppb)	Practical LOD (ppb)	Reliability (ppb)	Cost per Sample (USD)	Time per Sample (min)	Features	Ref.
NIPSOM	10	>20	Unreliable < 70	0.4	5	Colour sensitivity to yellow; working quickly.	[40,41]
Merck	10	>50	Unreliable < 70	0.5–1.00	30	Colour sensitivity to yellow; working quickly.	[40,41,42]
GPL	10	NA	Unreliable < 70	0.4	20	Colour sensitivity to yellow; working quickly.	[40]
AAIH and PH	50	>50	Unreliable < 70	0.4	NA	Colour sensitivity to yellow; working quickly.	[40,41]
AAN	10	>20	Unreliable < 70	0.4	30	Colour sensitivity to yellow; working quickly.	[40,41,42]
Quick As	5	NA	Can identify samples > 15	1.00–2.00	NA	Colour sensitivity to yellow; working quickly.	[43]
Hach Ez	10	NA	Can identify samples > 15	<1–2.00	20–40	Colour sensitivity to yellow; working quickly.	[42,43]
Arsenator	0.5–2	NA	Found to be correct 85% of the time, more reliable at lower concentrations	1.00	20	Ability to make accurate dilutions	[42,44]

**Table 2:** Some recent examples work on nanomaterials advancement in colorimetric detection of arsenic.

No	Metal Nanoparticles	Analyte	Detection Method	Limit of Detection (ppb)	Range of Detection (ppb)	Reference
1	Dithiothreitol coated Au-Nanorods	As(III)	UV-Vis spectrometry	10	10–100.1	[87]
2	AuNPs DNA aptamer	As(III)	UV-Vis spectrometry	161	76.6–766	[88]
3	GSH-functionalized AuNPs	As(III)	RGB extracting software	0.12	-	[70]
4	Glucose–AuNPs	As(III)	Naked eye	0.53	1–14	[89]
5	*Mangifera indica* leaf extract–AuNPs	As(III)	UV-Vis spectrometry	1.2	-	[90]
6	AuNPs-PEG	As(III)	UV-Vis spectrometry	5.0	-	[91]
7	Citrate-capped AuNPs	As(III)	UV-Vis spectrometry	1.8	4–100	[92]
8	Sucrose–AuNPs	As(III)	UV-Vis spectrometry	20	50–3000	[93]
9	GNP-MMT@Eu	As(III), As(V)	Naked eye	≤10.0	-	[74]
10	PEG–AgNPs	As(III)	UV–Vis spectrometry	1.0	5–13	[77]
11	Amino acid smart nanoprobe AuNPs	As(III)	UV–Vis spectrometry	0.22	1–200	[94]
12	ssDNA (Apt-21) AuNPs	As(III)	UV–Vis spectrometry	0.18	1–100	[95]
13	ss-AuNPs	As(III)	UV–Vis spectrometry	2.94	1–10	[96]
14	Ars-3 aptamer-CTAB-AuNPs	As(III)	UV-Vis spectrometry	16.9	1–100	[97]
15	RS-based Aptasensor with Aggregated AuNPs	As(III)	Naked eye	40	1–1500	[98]
16	GSH/DTT/Asn–AgNPs	As(III), As(V)	UV-Vis spectrometry	0.36	0.4–20	[71]
17	Ag-containing MOF	As(III)	Naked eye	50	n.a.	[84]
18	AgNPls-SiO_2_-Fh	As(III), As(V)	Naked eye	500	500–3000	[99]
19	Peptide–AuNPs	As(III), As(V)	Naked eye	1.54	n.a.	[100]
20	Asparagine–AuNPs	As(III)	UV-Vis spectrometry	100	100–2000	[101]
21	L-arginine-modified FeOOH	As(V)	UV-Vis spectrometry	0.42	0.67–3333.33	[102]
22	Oxidase-mimicking activity of Mn_3_O_4_ NPs	As(III), As(V)	Naked eye	1.32	5–1000	[103]
23	Cobalt oxyhydroxide (CoOOH) nanoflakes	As(V)	UV-Vis spectrometry	3.72	4–500	[104]
24	α-Fe_2_O_3_	As(V)	UV-Vis spectrometry	-	100–2000	[105]
25	DNA-functionalized Fe_3_O_4_ nanoparticles	As(V)	Fluorescence quenching	0.95	-	[106]
26	CuInS_2_ quantum dots@magnetic Fe_3_O_4_	As(V)	Fluorescence quenching	10	0.015–15,384.6	[107]
27	Iron oxyhydroxide (FeOOH) nanorods	As(V)	UV-Vis spectrometry	0.012	0.04–200	[108]
28	Co/Mo-MOF	As(V)	Naked eye	0.02	0.05–10^6^	[109]
29	Fe_3_O_4_ (core)-gold (shell)-thiol ligands	As(III)	UV-Vis spectrometry	0.86	-	[110]
30	Aptamer–As^III^ complex	As(III)	-	1.26	1.26–200	[111]
31	Dithiothreitol-capped Pd nanoparticles	As(III)	Naked eye	0.035	0.033–333.3	[112]
